# Supplementary material for: Modulating the Nature of Ionizable Lipids and Number of Layers in Hyaluronan-Decorated Lipid Nanoparticles for In Vitro Delivery of RNAi
Source: Pharmaceutics. 2024 Apr 20;16(4):563. doi: 10.3390/pharmaceutics16040563 (PMC11054633; doi:10.3390/pharmaceutics16040563)
Supplement: Supplementary file 1 [file pharmaceutics-16-00563-s001.zip › pharmaceutics-2924991-supplementary.pdf]

Supplementary Material

Modulating the Nature of Ionizable Lipids and Number of Layers in Hyaluronan-Decorated Lipid Nanoparticles for In Vitro Delivery of RNAi

Victor Passos Gibson, Houda Tahiri, Claudia Gilbert, Chun Yang, Quoc Thang Phan, Xavier Banquy, Pierre Hardy*

*** Correspondence:** Dr Pierre Hardy: pierre.hardy.med@ssss.gouv.qc.ca

1 Supplementary Figures

HA-LNPs DODMA 5x

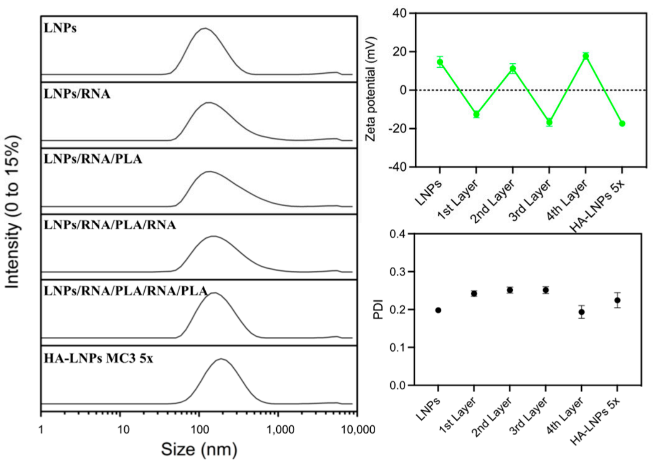

DLS measurement – HA-LNPs MC3				
	Z-ave (d.nm ± SD)	PDI (a.u. ± SD)	ζ-potential (mV ± SD)	**Encapsulation efficiency (%)**
LNPs (core)	119 ± 1	0.2 ± 0.01	15 ± 6	90 ± 1
LNPs/siRNA	149 ± 4	0.2 ± 0.01	-13 ± 1	N.D
LNPs/siRNA/PLA	160 ± 3	0.2 ± 0.01	11 ± 1	N.D
LNPs/siRNA/PLA/siRNA	159 ± 1	0.2 ± 0.01	-17 ± 2	N.D
LNPs/siRNA/PLA/siRNA/PLA	154 ± 1	0.2 ± 0.01	18 ± 1	N.D
HA-LNPs 5x	178 ± 1	0.2 ± 0.01	-17 ± 1	84 ± 1

Supplementary Figure S1. Physicochemical characterization of 5-layred HA-LNPs DODMA core. Left, intensity distribution of LNPs DODMA core (top graph) and after each polyelectrolyte layer. Middle, zeta potential (middle top, in green) and PDI (dot plot, black) of LNPs DODMA core before and after each polyelectrolyte layer. Right, top table represent the mean and SD of z-average, PDI, zeta potential at each step of the layer-by-layer technique. Encapsulation efficiency was determined for LNPs DODMA core with and without disruption of vesicles with triton (0.2%) and HA-LNPs 5x using SYBR Gold as intercalating agent. For HA-LNPs 5x, a solution of free siRNA was used for 100% fluorescence control. Right, bottom pictures, AFM of HA-LNPs DODMA core 5x.

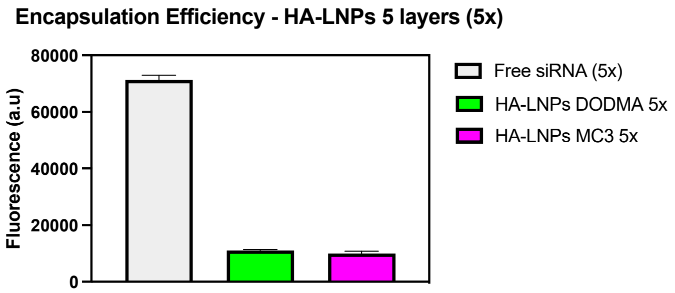

Supplementary Figure S2. Detailed fluorescence of free siRNA and layered-encapsulated siRNA in HA-LNPs 5x MC3 and DODMA core. Encapsulation efficiency measured by SYBR Gold intercalating reagent of siRNA located at the layer compartment for HA-LNPs DODMA (green bar) and HA-LNPs MC3 (magenta). Free siRNA (9.92 µM in a 38.1 µL solution) was used as 100% fluorescence control against aliquots of HA-LNPs. All conditions were diluted equally (80x) in 10 mM Tris/1mM EDTA buffer pH 7.4 and mixed with SYBR Gold 10X also diluted in TE Buffer.

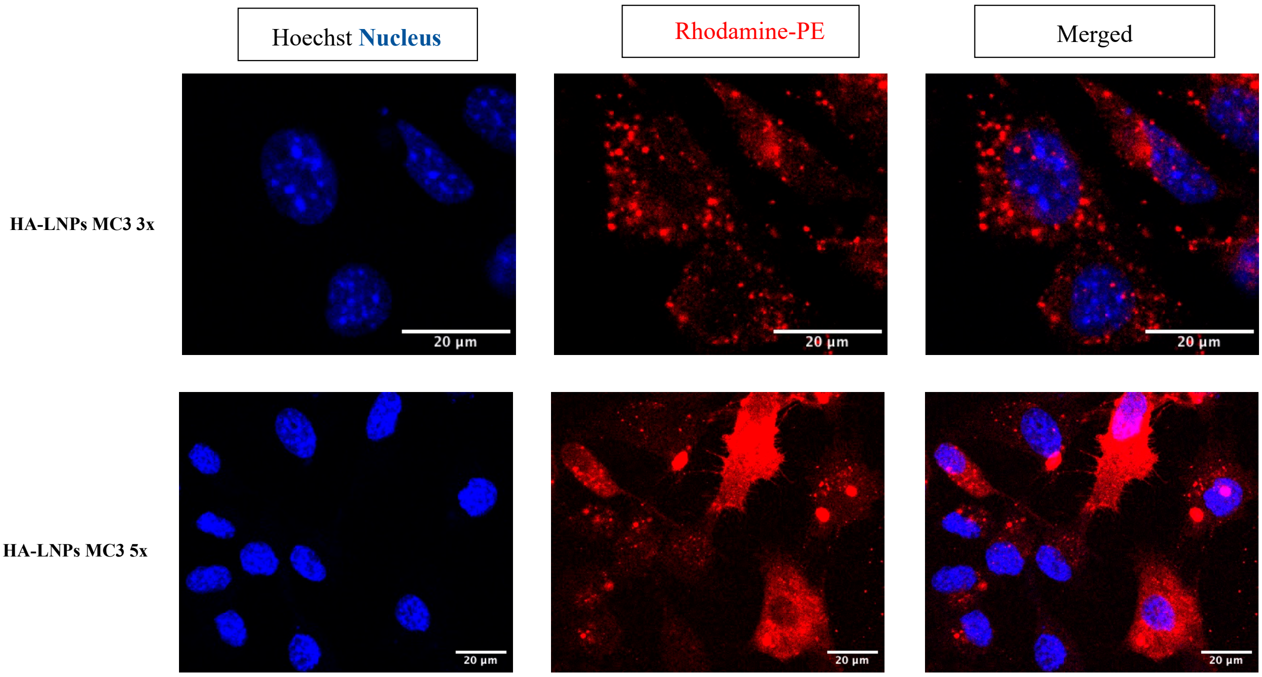

Supplementary Figure S3. Internalization of three (3x) or five-layered (5x) HA-LNPs MC3 core by GL261 cells. Rhodamine-PE at 0.1% mol ratio was used to formulate HA-LNPs MC3 core containing either 3 or 5 layers. Scramble siRNA was used for both core and layers. Particles were added at final concentration of 11 µM of total lipids per well and incubated overnight, followed by washing (PBS), fixation, a new round of washing and nuclei staining. Nuclei stained in blue (Hoechst 33,342) and particles in red (Rhodamine-PE). Scale bar: 20 µm for all images.

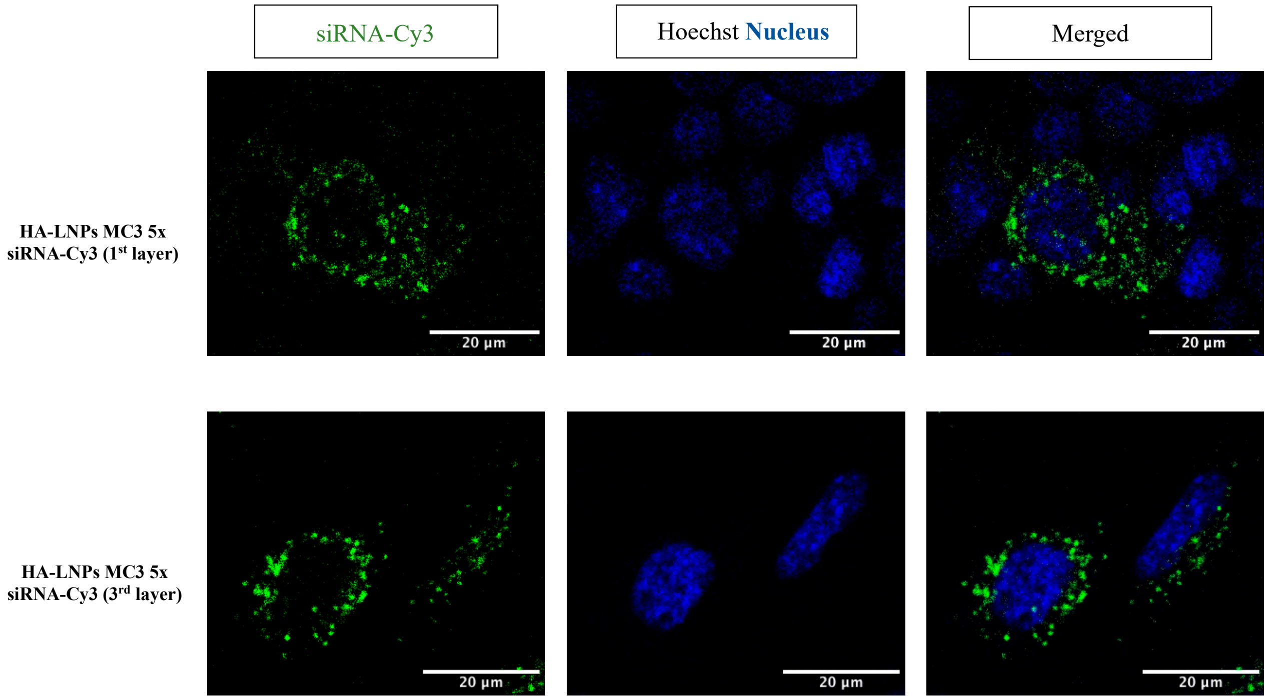

Supplementary Figure S4. Internalization five-layered (5x) HA-LNPs MC3 containing siRNA-Cyanine 3 at different layer location by GL261 cells. Cyanine-3-conjugated siRNA (siRNA-Cy3) was loaded either in the first or third layer of HA-LNPs MC3 5x. Scramble siRNA was used in the core and appropriate layer where siRNA-Cy3 was not located. Final concentration of siRNA-Cy3 was adjusted to 90 nM in all conditions. Particles were incubated overnight, washed, fixed, and stained with Hoechst 33,342 (blue, nuclei). Cyanine-3 is represented in green. Scale bar: 20 μm for all images.

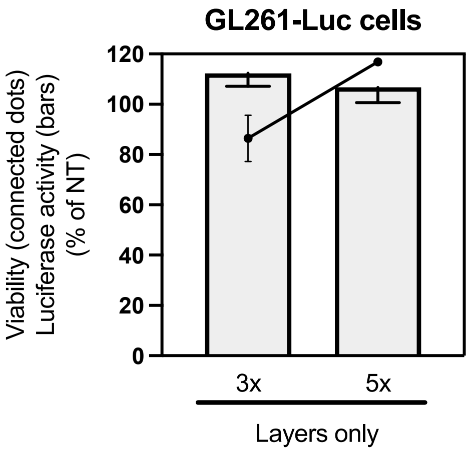

Supplementary Figure S5. Luciferase silencing in GL261-Luc cells (180 nM, 48h) mediated by layers component only equivalent to HA-LNPs 3x or 5x in the absence of LNPs core. Luciferase activity was measured 48h post transfection and reported as % of nontreated cells. The same for viability.
